# Correlations between plasma and PET beta-amyloid levels in individuals with subjective cognitive decline: the Fundació ACE Healthy Brain Initiative (FACEHBI)

**DOI:** 10.1186/s13195-018-0444-1

**Published:** 2018-11-29

**Authors:** Itziar de Rojas, J. Romero, O. Rodríguez-Gomez, P. Pesini, A. Sanabria, A. Pérez-Cordon, C. Abdelnour, I. Hernández, M. Rosende-Roca, A. Mauleón, L. Vargas, M. Alegret, A. Espinosa, G. Ortega, S. Gil, M. Guitart, A. Gailhajanet, M. A. Santos-Santos, Sonia Moreno-Grau, O. Sotolongo-Grau, S. Ruiz, L. Montrreal, E. Martín, E. Pelejà, F. Lomeña, F. Campos, A. Vivas, M. Gómez-Chiari, M. A. Tejero, J. Giménez, V. Pérez-Grijalba, G. M. Marquié, G. Monté-Rubio, S. Valero, A. Orellana, L. Tárraga, M. Sarasa, A. Ruiz, M. Boada, on behalf of the FACEHBI study, C. Abdelnour, N. Aguilera, M. Alegret, M. Berthier, M. Boada, M. Buendia, S. Bullich, F. Campos, P. Cañabate, C. Cuevas, I. de Rojas, A. Espinosa, A. Gailhajenet, S. Diego, S. Gil, J. Giménez, R. Gismondi, M. Gómez-Chiari, M. Guitart, I. Hernández, M. Ibarria, A. Lafuente, F. Lomeña, M. Marquié, E. Martín, J. Martínez, A. Mauleón, G. Monté, M. Moreno, S. Moreno-Grau, L. Núñez, A. Orellana, G. Ortega, A. Páez, A. Pancho, J. Pavía, E. Pelejà, A. Pérez-Cordon, V. Pérez-Grijalba, P. Pesini, S. Preckler, O. Rodríguez-Gómez, J. Romero, M. Rosende-Roca, A. Ruiz, S. Ruiz, L. Montrreal, A. Sanabria, M. A. Santos-Santos, M. Sarasa, O. Sotolongo-Grau, L. Tárraga, M. A. Tejero, M. Torres, S. Valero, L. Vargas, A. Vivas

**Affiliations:** 1 Research Center and Memory Clinic, Fundació ACE, Institut Català de Neurociències Aplicades, Universitat Internacional de Catalunya-Barcelona, C/ Marquès de Sentmenat, 57, 08029 Barcelona, Spain; 2 Araclon Biotech©, Zaragoza, Spain; 3 Servei de Medicina Nuclear, Hospital Clínic i Provincial, Barcelona, Spain; 4 Departament de Diagnòstic per la Imatge, Clínica Corachan, Barcelona, Spain

**Keywords:** Subjective cognitive decline, Preclinical AD, Alzheimer’s disease, Amyloid β, Plasma biomarker, TP42/40, PET, Florbetaben

## Abstract

**Background:**

Peripheral biomarkers that identify individuals at risk of developing Alzheimer’s disease (AD) or predicting high amyloid beta (Aβ) brain burden would be highly valuable. To facilitate clinical trials of disease-modifying therapies, plasma concentrations of Aβ species are good candidates for peripheral AD biomarkers, but studies to date have generated conflicting results.

**Methods:**

The Fundació ACE Healthy Brain Initiative (FACEHBI) study uses a convenience sample of 200 individuals diagnosed with subjective cognitive decline (SCD) at the Fundació ACE (Barcelona, Spain) who underwent amyloid florbetaben(^18^F) (FBB) positron emission tomography (PET) brain imaging. Baseline plasma samples from FACEHBI subjects (aged 65.9 ± 7.2 years) were analyzed using the ABtest (Araclon Biotech). This test directly determines the free plasma (FP) and total plasma (TP) levels of Aβ40 and Aβ42 peptides. The association between Aβ40 and Aβ42 plasma levels and FBB-PET global standardized uptake value ratio (SUVR) was determined using correlations and linear regression-based methods. The effect of the *APOE* genotype on plasma Aβ levels and FBB-PET was also assessed. Finally, various models including different combinations of demographics, genetics, and Aβ plasma levels were constructed using logistic regression and area under the receiver operating characteristic curve (AUROC) analyses to evaluate their ability for discriminating which subjects presented brain amyloidosis.

**Results:**

FBB-PET global SUVR correlated weakly but significantly with Aβ42/40 plasma ratios. For TP42/40, this observation persisted after controlling for age and *APOE* ε4 allele carrier status (*R*^2^ = 0.193, *p* = 1.01E-09). The ROC curve demonstrated that plasma Aβ measurements are not superior to *APOE* and age in combination in predicting brain amyloidosis. It is noteworthy that using a simple preselection tool (the TP42/40 ratio with an empirical cut-off value of 0.08) optimizes the sensitivity and reduces the number of individuals subjected to Aβ FBB-PET scanners to 52.8%. No significant dependency was observed between *APOE* genotype and plasma Aβ measurements (*p* value for interaction = 0.105).

**Conclusion:**

Brain and plasma Aβ levels are partially correlated in individuals diagnosed with SCD. Aβ plasma measurements, particularly the TP42/40 ratio, could generate a new recruitment strategy independent of the *APOE* genotype that would improve identification of SCD subjects with brain amyloidosis and reduce the rate of screening failures in preclinical AD studies. Independent replication of these findings is warranted.

**Electronic supplementary material:**

The online version of this article (10.1186/s13195-018-0444-1) contains supplementary material, which is available to authorized users.

## Highlights

- Brain and plasma Aβ levels are partially correlated in SCD subjects.
- Plasma Aβ measurements are independent of *APOE* genotype.
- The model including only plasma TP42/40 level as a variable achieved the highest sensitivity in predicting Aβ PET positivity (83%).
- A simple preselection step using the TP42/40 classifier with an empirical cut-off value of 0.08 would reduce the number of individuals subjected to Aβ FBB-PET by 52.8%.

## Background

Alzheimer’s disease (AD), the most common cause of dementia, is a neurodegenerative disorder characterized by progressive memory loss and cognitive decline [1]. Pathological findings of AD include deposits of amyloid beta (Aβ) peptides in the brain conforming extracellular amyloid plaques together with intracellular deposits of hyperphosphorylated tau [2]. The progressive increase of both pathological hallmarks is associated with gradual synaptic and neuronal loss resulting in the clinical deterioration of patients [3].

There are no effective disease-modifying therapies for AD available at the current time. Neuropsychological assessment [4], cerebrospinal fluid [5] (CSF) analysis, and amyloid positron emission tomography (PET) scans are common methods used for prodromal AD detection. CSF and amyloid PET provide the most reliable in-vivo biomarkers of prodromal AD, but they are not suitable for population screening purposes due to the invasive CSF sampling procedure and the high cost and limited availability of amyloid PET imaging [6, 7]. Magnetic resonance imaging (MRI)-based AD biomarkers have demonstrated high sensitivity to prodromal AD [8]; however, the specificity of MRI is limited for predicting conversion of mild cognitive impairment (MCI) to dementia [9] and MRI is also impractical in patients with some types of pacemakers, metal implants, or claustrophobia. Consequently, despite the robustness of these biomarkers, they are not suitable for broad population screening in primary care clinical settings. Therefore, there is a growing need for accurate identification of asymptomatic (preclinical) individuals with underlying AD pathology to improve diagnosis and subject inclusion in prevention trials of prodromal and presymptomatic AD.

Discovery of blood-based AD biomarkers would entail important cost-benefit and scalability advantages over current techniques, potentially enabling broader clinical access and efficient population screening. The plasma concentration of Aβ is a logical candidate, but studies to date have produced conflicting results on its utility [10]. Several longitudinal studies with large cohorts such as the Framingham Study [11] with 2189 dementia-free participants followed from baseline until they developed dementia, died, or had been followed for 10 years and the Rotterdam Study [12] with 1756 participants and 392 incident dementia cases identified (follow-up mean 8.6 years) have reported increased risk of dementia associated with lower Aβ42/40 plasma ratios and that a reduction in plasma Aβ42 levels over time is linked with cognitive decline [13, 14]. A recent publication [15] studied the ability of Aβ precursor protein (APP/Aβ42), Aβ40/Aβ42 ratios, and their composites to predict individual brain Aβ^+/−^ status determined by Aβ-PET imaging. The results showed that all test biomarkers correlated with both Aβ PET burden and levels of Aβ42 in CSF in two independent cohorts, demonstrating that the three different types of Aβ-related biomarkers (plasma, CSF, and PET imaging) are highly correlated with each other, clearly indicating the potential utility of plasma biomarkers. Furthermore, an independent study [16] suggests that individuals with subjective cognitive decline (SCD) exhibit significantly higher Aβ42 plasma concentrations compared with participants with no complaints. However, other studies have reported a weak or even a lack of association of plasma Aβ42/40 ratio with AD diagnosis [17–19].

Given that both subjective complaints and impaired episodic memory are present in MCI, the existence of an earlier distinct clinical stage where subjective complaints exist in the absence of detectable objective cognitive deficits is plausible [20]. There is evidence suggesting that SCD may increase the risk of progression to cognitive impairment and dementia [21], and that individuals with SCD have a higher risk of developing AD [22], and present more functional deficits [23] and AD brain pathology than non-SCD participants [24]. SCD might represent the earliest point on the continuum of clinical Alzheimer’s symptomatology [25–27], even anticipating the onset of subtle but detectable neuropsychological or biological alterations. Hence, a better understanding of the baseline characteristics of this group of patients may enhance our knowledge of early AD processes, facilitating early diagnosis, follow-up, and preventive treatment, making SCD an interesting target population to study.

The primary aim of this study was to assess the association between plasma Aβ levels and amyloid brain burden. Specifically, we measured Aβ42 and Aβ40 plasma levels using two specific sandwich enzyme-linked immunosorbent assay (ELISA) kits, ABtest40 and ABtest42 (Araclon Biotech, Zaragoza, Spain), and quantified amyloid brain burden using florbetaben(^18^F) (FBB)-PET global standardized uptake value ratio (SUVR) in 200 individuals with SCD. We evaluated whether plasma Aβ ratios may be useful biomarkers for AD and a screening tool for amyloidosis in healthy populations.

## Methods

### The FACEHBI cohort

The Fundació ACE Healthy Brain Initiative (FACEHBI) uses a convenience sample of 200 individuals (mean age 65.8 ± 7.2 years; 37.5% males) diagnosed with SCD at Fundació ACE (Barcelona, Spain) recruited from Open House initiatives [28]. The cohort comprised of 52 (26%) *APOE* ε4 allele carriers and 18 (9%) individuals with a positive (SUVR > 1.45) FBB-PET scan. The demographic characteristics of the study cohort are summarized in Table 1 and Additional file 1 (Table S1) by FBB-PET status.

**Table 1:** Demographics and clinical characteristics of the study cohort (FACEHBI [29])

Variable	SCD
Subjects, *n*	200
Age, years	65.87 (7.23)
Education, years	14.76 (4.73)
Gender, % males	37.5
*APOE*, % e4 allele carriers	26
Creatinine, mg/dl	0.92 (0.15)
Body mass index, kg/m^2^	26.64 (4.32)
Hematocrit, %	43.15 (4.93)
FBB-PET SUVR	1.2 (0.15)
FP42/40	0.04 (0.03)
TP42/40	0.09 (0.06)
FP40/TP40	0.44 (0.06)
BP42/40	0.13 (0.09)
FP42/TP42	0.24 (0.21)

Data are shown as mean (SD) unless otherwise specified

*APOE* apolipoprotein, *BP* bound peptide, *FBB* florbetaben(^18^F), *FP* free plasma, *PET* positron emission tomography, *SCD* subjective cognitive decline, *SUVR* standardized uptake value ratio, *TP* total plasma

The SCD criteria used to recruit subjects in this study have been described previously [29]. Briefly, inclusion criteria were: 1) subjective cognitive complaints defined as a score of ≥ 8 on MFE-30, the Spanish version of the Memory Failures in Everyday Life Questionnaire [30]; 2) Mini-Mental State Examination (MMSE) score ≥ 27; 3) Clinical Dementia Rating (CDR) = 0; and 4) performance on the Fundació ACE Neuropsychological Battery (NBACE) [31] within the normal range for age and educational level. Exclusion criteria were as follows: 1) relevant symptoms of anxiety or depression defined as a score of ≥ 11 on the Hospital Anxiety and Depression Scale (HADS) [32]; 2) presence of other psychiatric diagnosis; 3) history of alcoholism and epilepsy; and 4) known renal or liver failure.

Cognitive assessment was performed according to the routines of the Memory Clinic of Fundació ACE as described elsewhere [33]. Baseline MRI of these subjects demonstrated the absence of signs indicative of brain pathology. All participants gave written consent and the protocol was approved by the ethics committee of the Hospital Clinic i Provincial (Barcelona, Spain) (EudraCT: 2014–000798-38).

### MRI acquisition

All MRI scans were acquired prior to FBB-PET. MRI were performed on a 1.5-T Siemens Magneton Aera (Erlangen, Germany) using a 32-channel head coil. Anatomical T1-weighted images were acquired using a rapid acquisition gradient-echo three-dimensional (3D) magnetization-prepared rapid gradient-echo (MPRAGE) sequence with the following parameters: repetition time (TR) 2.200 ms, echo time (TE) 2.66 ms, inversion time (TI) 900 ms, slip angle 8°, field of view (FOV) 250 mm, slice thickness 1 mm, and isotropic voxel size 1 × 1 × 1 mm. Subjects also received axial T2-weighted, 3D isotropic fast fluid-attenuated inversion recovery (FLAIR) and axial T2*-weighted sequences to detect significant vascular pathology or microbleeds.

### FBB-PET acquisition

FBB-PET scans were obtained with a Siemens© Biograph molecular-CT machine. PET images were acquired in 20 min starting from 90 min after intravenous administration of 300 Mbq of Florbetaben(^18^F) radio tracer (NeuraCeq©), administered as a single slow intravenous bolus (6 s/ml) in a total volume of up to 10 ml.

#### SUVR estimation

MRI cortical [34] and subcortical [35] parcellations were carried out with Freesurfer 5.3 (http://surfer.nmr.mgh.harvard.edu/), following the pipeline described in https://surfer.nmr.mgh.harvard.edu/fswiki/recon-all.

FBB-PET were coregistered to the MRI labeled data with the FSL 5.0 software package (https://fsl.fmrib.ox.ac.uk/fsl/fslwiki) by means of MCFLIRT, it is an intra-modal motion correction tool based on optimization and registration techniques from FLIRT (FMRIB's Linear Image Registration Tool), which next was also used. These are fully automated tools implemented in FSL 5.0 for linear (affine) intra- and inter-modal brain image registration [36, 37].

Amyloid cortical SUVR was determined as the average of the standardized uptake value normalized by the uptake in the cerebellar grey matter, with this reference region being selected from the MRI cerebellum external and cortex segments. Based on previous studies [38], a cut-off for SUVR above or equal to 1.45 was selected as the amyloid positivity criterion.

### Blood sampling, APOE genotyping, biochemical determinations, and Aβ measurements

Blood samples and the *APOE* genotype from each participant were routinely processed in Fundació ACE as previously described [29, 39]. In brief, blood samples were obtained in the morning after an overnight fast, collected in polypropylene vials with ethylenediaminetetraacetic acid (EDTA) and immediately refrigerated. Samples were centrifuged within 24 h from extraction to collect the plasma and then aliquoted and frozen at −80 °C until assayed. Biochemical and hematologic measurements were determined in a reference laboratory according to clinical standards.

For plasma amyloid testing, four determinations were made (Additional file 2). Total plasma (TP) and free plasma (FP) Aβ40 and Aβ42 levels were quantified using two specific sandwich ELISA kits, Aβtest40 and Aβtest42 (Araclon Biotech, Zaragoza, Spain), in accordance with the manufacturer’s instructions as described elsewhere [39]. Briefly, before analysis, each plasma sample was split into two aliquots: an undiluted aliquot and another aliquot pretreated by 1:3 dilutions in a formulated sample buffer (phosphate-buffered saline (PBS) 0.5 M, 0.5% Tween-20, 1% blocking polymer) intended to break Aβ interactions with other plasma components. Thus, levels of free and total Aβ40 and Aβ42 were separately determined in undiluted and diluted plasma, respectively. The difference between TP and FP concentration corresponds to the amount of amyloid peptide bound to plasma components (BP). The Aβ42/Aβ40 ratios in each of these plasma fractions (FP42/40, TP42/40, BP42/40, FP40/TP40, and FP42/TP42) were calculated and served as the target plasma biomarkers for this study.

The levels of TP and FP obtained from plasma samples were expressed as picograms (pg) of Aβ peptide per milliliter (ml) of plasma. The analyses were always performed in duplicates of the same aliquot and in a coded manner to ensure blindness of the operator.

Both inter-assay and intra-assay coefficients of variation were below 5% and 8–20% for ABtest40 and ABtest42, respectively. The detection limit was 3.13 and 200 pg/ml for ABtest40 and 1.56 and 100 pg/ml for ABtest42. One sample was removed from the original FACEHBI cohort [29] because both ABtest determinations were outside the upper limit of quantification (> ULQ). In ABtest42, 84 of 400 (21%) determinations were also outside the quantification range, either because they were below the lower limit of quantification (< LLQ) or due to undetectable peptide levels. We assigned the minimum value of quantification (1.56 pg/ml) to these samples.

### Statistical analysis

We performed several correlation and regression analyses to explore the association between plasma amyloid ratios and FBB-PET brain amyloid burden. First, we conducted a linear regression analysis using FBB-PET global SUVR as the quantitative response variable in SCD subjects. FBB-PET global SUVR was log-transformed for all analyses since it was not normally distributed. The distribution of variables and Shapiro-Wilk test are given in Additional file 3 (Figure S1). We conducted an exploratory analysis with three different transformations for the plasma Aβ42/Aβ40 ratios: dichotomous (with regard to the median of the population), quartile, and logarithmic. First, we performed Pearson and Spearman correlation analyses between log-transformed FBB-PET global SUVR and the raw values of each plasma Aβ measure of interest as well as the transformed plasma Aβ ratios (Table 2 and Additional file 4: Table S2). Next, we performed a linear regression analysis using a backward-selection procedure with FBB-PET global SUVR as the quantitative dependent variable, with age, gender, education, *APOE* ε4 carrier status, and the best performing log-transformed plasma Aβ42/40 ratio as independent variables (Table 3 and Additional file 5: Table S3). Bonferroni correction was used to adjust for multiple comparisons.

**Table 2:** Correlation between direct Aβ plasma and log-transformed FBB-PET SUVR

Logarithmic				
L_PET	FP42/40	TP42/40	FP42/TP42	FP40/TP40
Pearson’s *r* (*n* = 199)	−0.160*	−0.248**	0.100	0.085
*p* value (2-tailed)	0.024	4.04E-04	0.162	0.231
95% confidence interval	−0.292 to −0.021	−0.374 to −0.113	− 0.04 to 0.236	−0.055 to 0.221

Plasma amyloid beta (Aβ)42/40 ratios were transformed in logarithmic scale

Bonferroni correction was used to adjust for multiple comparisons (< 1.92E-03)

*FP* free plasma, *L_PET*, logarithmic transformed positron emission tomography score, *TP* total plasma

*p ≤ 0.05

**p ≤ 0.01

**Table 3:** Backward selection regression analysis: amyloid beta plasma TP42/40 ratio and log FBB-PET global SUVR with covariates

	Estimate	Standard error	T value	*p* value
(Intercept)	−0.0701	0.030	−2.301	0.022*
Age	0.0015	4.45E-04	3.573	4.45E-04***
*APOE*	0.035	0.007	5.107	7.75E-07***
Log TP42/40	−0.041	0.011	−3.794	1.98E-04***

Residual standard error = 0.042 on 195 degrees of freedom (DF)

Adjusted *R*^2^ = 0.193; F = 16.75 on 3 and 195 DF; *p* value = 1.01E-09

Backward selection regression analysis adjusting for age, *APOE* and TP42/40; statistical significance was set to *p* < 1.92E-03 after Bonferroni correction for multiple comparisons

*APOE* apolipoprotein, *FBB* florbetaben(^18^F), *PET* positron emission tomography, *SUVR* standardized uptake value ratio, *TP* total plasma

*p ≤ 0.05

***p < 0.001

We used logistic regression to construct four different models (Table 4) to evaluate the usefulness of the covariates selected from the backward regression model for discriminating which SCD participants were FBB-PET amyloid positive (> 1.45) in 199 participants. The models were structured to reflect categories of predictive information by the ease of its acquisition. Accordingly, the first model (model #1) included only predictors that can be easily obtained (age). The second model additionally requires a blood extraction and includes two parts: model #2a for *APOE* ε4 carrier status (0–1) which served as the reference model for discrimination of amyloid PET-positive subjects as proposed by Petersen [25], and model #2b for a plasma determination of TP42/40 in log units. The third model (model #3) included the three variables described above (age, *APOE*, and TP42/40). Finally, the fourth model (model #4) only included the target plasma biomarker (logTP42/40). We used the area under the receiver operating characteristic curve (AUROC) from the models as a measure of how well the model discriminated between FBB-PET positive and negative subjects. The criterion for choosing the operating point along the ROC curve was Youden’s index maximum. The logistic models allowed us to assign a predicted probability of being FBB-PET SUVR positive to each subject based on values for the selected variables in the model. In addition to sensitivity/specificity performance measures, the predictive values (positive (PPV) and negative (NPV)) of the models were calculated.

**Table 4:** Summary of logistic regression models and AUROC analysis

Characteristic	Model 1		Model 2a		Model 2b		Model 3		Model 4	
	OR (95% CI)	*p*	OR (95% CI)	*p*	OR (95% CI)	*p*	OR (95% CI)	*p*	OR (95% CI)	*p*
Age	1.091 (1.016–1.172)	0.017	1.114 (1.033–1.202)	0.005	1.097 (1.017–1.183)	0.017	1.113 (1.029–1.205)	0.008	–	–
*APOE*	–	–	7.319 (2.503–21.401)	2.8E-04	–	–	7.208 (2.431–21.373)	3.7E-4	–	–
Log TP42/40	–	–	–	–	0.131 (0.021–0.819)	0.030	0.126 (0.017–0.944)	0.044	0.133 (0.021–0.829)	0.031
AUROC	0.702		0.806		0.754		0.818		0.681	
Youden’s index	0.42		0.54		0.49		0.55		0.42	
Cut-off	0.101		0.147		0.115		0.092		0.081	
Specificity	69.6		87.3		76.8		77.3		59.1	
Sensitivity	72.2		66.7		72.2		77.8		83.3	
PPV	19.1		34.3		23.6		25.5		16.7	
NPV	96.2		96.3		96.5		97.2		97.2	

Logistic regression models were used to assess predictors of FBB-PET SUVR positivity (cut-off > 1.45) after adjustment by selected covariates in 199 participants

Model 1 included only age as a predictor; model 2 requires a blood extraction (*APOE* ε4 carrier status (0–1) or log TP42/40, model 2a and 2b respectively); model 3 included age, *APOE*, and log TP42/40, and model 4 only included the target plasma biomarker log TP42/40

The criterion for choosing the operating point along the ROC curve was Youden’s index maximum

*AUROC* area under the receiver operating characteristic curve, *CI* confidence interval, *NPV* negative predictive value, *OR* odds ratio, *PPV* positive predictive value, *TP* total plasma

Finally, the effect of *APOE* genotype on plasma Aβ levels was assessed by comparing Aβ plasma measurements between *APOE* ε4 carriers and noncarriers by analysis of variance (ANOVA) (Additional file 6: Table S4) by performing separate regression analyses between logTP42/40 and FBB-PET global SUVR in *APOE* ε4 carriers and noncarriers (Additional file 7: Figure S4), and by testing the interaction term between *APOE* ε4 carrier status and logTP42/40 in the logistic regression model #3 described above. Statistical analysis was performed with SPSS 19 and RStudio Version 1.0.136. The Ggplot2 package was used for graphic representations.

## Results

### Relationship between Aβ plasma ratio and FBB-PET

The FACEHBI study has been designed to identify the most important factors related to preclinical AD [29]. To evaluate the strength of the association between plasma amyloid biomarkers and Aβ-PET burden, we conducted correlation analyses. Logarithmic TP42/40 and FP42/40 showed significant negative Pearson’s correlations with amyloid PET burden, although only TP42/40 exceeds the Bonferroni correction (*r* = −0.248 (−0.374 to −0.113); *p* = 4.04E-04). In contrast, direct plasma levels of Aβ40 and Aβ42 did not significantly correlate with FBB-PET global SUVR (Additional file 4: Table S2C). BP42/40 was excluded from further analyses due to collinearity with TP42/40 (Pearson’s *r* = 0.972 (0.963–0.979); *p* < 2.2E-16; Additional file 4: Table S2A).

**Fig. 1:**
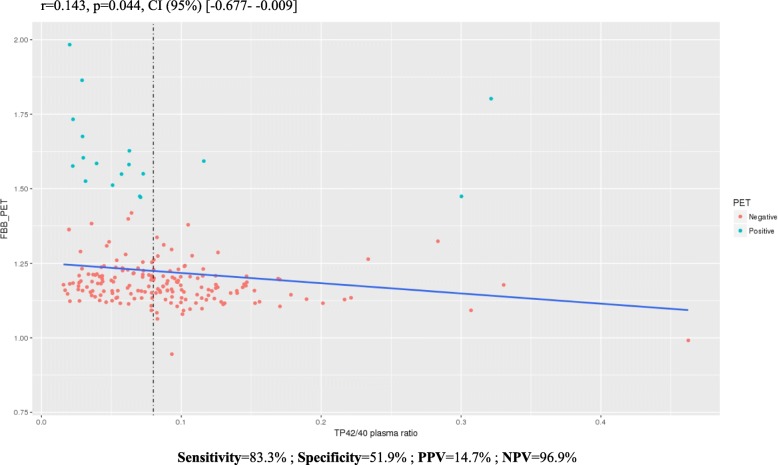
Linear regression between florbetaben(^18^F) (FBB)-positron emission tomography (PET) global SUVR and Aβ total plasma (TP)42/40 plasma ratio in SCD subjects. Inverse association between Aβ TP42/40 plasma ratio and FBB-PET scan. Experimental cut-off point of Aβ plasma ratio TP42/40 established at 0.08 to reduce the prescreening number of Aβ FBB-PET scans to 52.8%. CI confidence interval, NPV negative predictive value, PPV positive predictive value

Backward regression analysis identified age, *APOE* ε4 status (0–1), and logTP42/40 as significant covariates of the best model predicting FBB-PET global SUVR (*R*^2^ = 0.193 and *p* value = 1.01E-09; Table 3). The inverse association between FBB-PET SUVR and TP42/40 is graphically represented with raw data in Fig. 1. The associations with the other Aβ plasma biomarkers are shown in Additional file 8 (Figure S2). After stratifying for *APOE* ε4, the linear regression analysis showed a negative relationship between plasma TP42/40 and FBB-PET uptake (*r* = −0.523 (−0.185 to −0.067); *p* = 8.12E-05) exclusively in *APOE* ε4 carriers (Additional file 9: Figure S3).

**Fig. 2:**
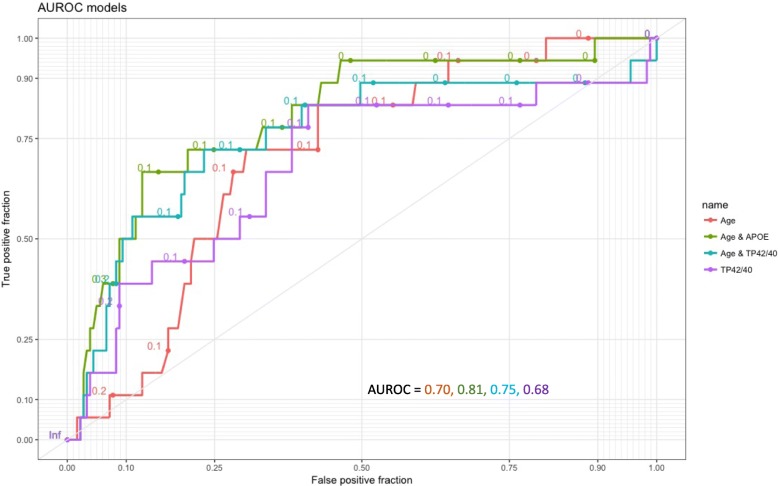
Area under the receiver operating characteristic curve (AUROC) models. AUROC analysis evaluated the discrimination between FBB-PET SUVR positive and negative subjects in different models from Table 4. APOE apolipoprotein E, TP total plasma

To assess the relevance of the plasma biomarkers in predicting amyloid PET positivity, the TP42/40 model was selected for the subsequent AUROC analysis. Education and gender were excluded due to their lack of significance in the backward regression model. When Aβ-PET was used as the standard classifier for Aβ^+^/Aβ^–^ status, all models worked in a similar way to the reference discrimination model #2a with age and *APOE* as predictors (AUROCs of 0.702, 0.806, 0.754, 0.818, and 0.681 for models #1, #2a, #2b, #3, and #4 respectively; Table 4, Fig. 2). The effect and significance of TP42/40 was maintained in the different models indicating a robust association with Aβ-PET positivity. Model #2a presented the best balance between PPV/NPV (34.3–96.3%, respectively), but at the same time showed the lowest sensitivity (66.7%). On the other hand, TP42/40 alone (model #4) achieved the best sensitivity (83.3%) and a good NPV (97.2%), indicating its value as a potential screening tool for detecting brain amyloidosis (Table 4). Using an empirical cut-off point of TP42/40 = 0.08, individuals with a TP42/40 plasma ratio < 0.08 (52.8%) would be prescreened with a FBB-PET scan, capturing 83% of the positive amyloid cases, thus reducing the prescreening number of Aβ FBB-PET (sensitivity = 83.3%; specificity = 51.9%; NPV = 96.9%; PPV = 14.7%; Fig. 1).

### Effect of APOE genotype on plasma Aβ levels

In the current study, we found no association between *APOE* genotype and plasma Aβ measurements, indicating independence between both variables. No plasma Aβ measure significantly differed between *APOE* ε4 carriers and noncarriers (Additional file 6: Table S4 and Additional file 7: Figure 4). This independence, confirmed by the absence of significance for the interaction term between *APOE* ε4 and logTP42/40 (Additional file 10: Table S5) (odds ratio (OR) 0.022, 95% confidence interval (CI) 2.30E-04 to 2.201); *p* = 0.105) could be an advantage if using this biomarker as a screening tool since it would avoid bias resulting from *APOE* screening.

## Discussion

The FACEHBI study has been designed to identify the most relevant factors related to preclinical AD in a cohort of individuals with SCD [29]. FACEHBI has a 9% prevalence of amyloid PET positivity, which is lower than similar series reported in the literature. Ossenkoppele et al. [40] estimated a prevalence of 11% brain amyloid positivity in a cohort of healthy controls aged 55–64 years, and 22% in those aged 65–74 years. In a meta-analysis [41], Jansen et al. reported a prevalence of amyloid PET positivity of approximately 20% at age 65 years. The Mayo Clinic population study [42] showed a prevalence of amyloid PET positivity of 13% in the age group 60–64 years and 32% in those aged 65 to 69 years. A possible cause for the low prevalence of amyloid PET positivity in the FACEHBI cohort is that a strict definition of cognitive normality was used. A score of 1.5 SD below the mean according to age and level of education in any single NBACE [43] test precluded individuals from enrolling into the FACEHBI study. Other studies with a more liberal definition of cognitive normality included patients that would have been considered to have MCI by our standards, presumably increasing their prevalence of amyloid PET positivity. Secondly, the setting of the study is relevant, as it is well known that participants from clinical samples tend to show higher risk of cognitive progression (and probably greater brain amyloidosis) than those from population-based samples and healthy volunteers, even though both groups are considered to be cognitively normal. In this regard, FACEHBI is a mixed sample, but most of our participants (70%) are healthy volunteers from the community who came to check their cognition for free through Open Door Initiatives. This could partly explain a lower prevalence of brain amyloidosis in our FACEHBI participants compared with pure clinical samples.

The main finding of this study is that lower plasma Aβ42/40 ratios (particularly the TP42/40 ratio) correlate with higher cerebral Aβ plaque burden assessed by amyloid FBB-PET imaging in the FACEHBI SCD cohort. This inverse correlation is presumably driven by the reduction of Aβ42 and the increase of Aβ40 in the Aβ^+^ population (Additional file 11: Figure S5). These results are independent of previous explorations and are in line with other promising results reporting similar associations between plasma Aβ42/40 ratio and cortical fibrillary Aβ burden [15, 44–50] (for review see [51–53]). This study provides added value as it is one of few [48, 49] that explores the association between Aβ plasma ratios and Aβ brain burden within a population of cognitively normal individuals, avoiding the possibility of potential circular associations related to inclusion of MCI and AD subjects along with healthy controls in the same models. Nevertheless, discrepant results from other studies [17–19, 54–56] that assessed the performance of plasma Aβ levels in predicting the Aβ brain status cannot be disregarded. Part of this controversy could be explained by the mixed distribution of individuals with and without cerebral Aβ deposition (as quantified by amyloid PET and/or by CSF analysis) among healthy controls, MCI, and demented individuals.

It is believed that the clearance of brain Aβ is reduced in AD patients compared with healthy controls. This is consistent with a report by Giedraitis et al. [57] who found a correlation between CSF and plasma levels of both Aβ40 and Aβ42 in healthy individuals, whereas no correlations were seen in AD or MCI patients. Thus, the search for an association between blood and brain Aβ levels should be directed towards the earliest stages of the disease (preclinical/prodromal AD), which is also when it is of maximum clinical interest especially as a target population for the development of novel disease-modifying therapies. However, it has been difficult to draw definite conclusions with respect to changes in plasma Aβ concentration in AD [52] because of the inconsistency of the available data. Stringent standardization is required to obtain reliable data that facilitate comparison between studies. In this study we used Aβ42/Aβ40 plasma ratios (particularly the TP42/40 ratio) instead of single peptide measurements to attenuate possible bias in single Aβ peptide level quantifications caused by inconsistencies in sample handling [58].

The regression model that included only the TP42/40 ratio did not show sufficient predictive ability to identify those individuals with a positive FBB-PET scan, accounting for only 20% of the variance. Clearly, screening with these factors would not be an acceptable option for determining amyloid PET positivity in the clinical practice setting. Nevertheless, the plasma TP42/40 ratio showed a significant negative correlation with FBB-PET SUVR. This suggests that this plasma Aβ biomarker could be useful as an enrichment tool to identify potential candidates for clinical trials focused on preclinical AD. To prove this, we would need to reproduce the results in a controlled trial with an independent sample. Our analyses suggest that inclusion of the TP42/40 plasma biomarker in a classifier model could reduce unnecessary amyloid PET scans, facilitating recruitment for clinical trials. Taking this into account, in a clinical trial recruiting scenario targeting cognitively normal people, a prescreening step using a TP42/40 classifier (cut-off value = 0.08) would reduce the number of individuals undergoing Aβ FBB-PET scans to 52.8%. The cortical Aβ burden of these subjects would have to then be confirmed by Aβ FBB-PET scans. Consequently, this strategy would reduce the costs [59] of identifying individuals with brain amyloidosis for AD prevention trials [60].

We observed an association of age with plasma Aβ ratios as described in previous studies [41, 42, 59, 61]. No association was found between the *APOE* ε4 genotype and Aβ plasma ratios, demonstrating independence between *APOE* ε4 genotype and this candidate plasma biomarker. The linear regression analysis stratifying for *APOE* ε4 showed a negative relationship between TP42/40 and FBB-PET SUVR in *APOE* ε4 carriers but not in noncarrier SCD individuals. At first glance, these results seem contradictory with other studies reporting a significant negative relationship between plasma Aβ and amyloid PET only in *APOE* ε4 noncarriers [46, 48, 62, 63]. One possible explanation could stem from the difference in cohort composition, as the previous studies included patients with MCI and AD diagnosis, while our sample is comprised only of SCD individuals. Therefore, their *APOE* ε4 carrier group included participants who were older and more cognitively impaired than ours, whereas their *APOE* ε4 noncarrier group could be more similar to our *APOE* ε4 carrier group in terms of demographics and cognition. Therefore, they observed a negative correlation between Aβ plasma and PET in *APOE* ε4 noncarriers that would be equivalent to the correlation in *APOE* ε4 carriers in our study. We attribute this finding to the potential enrichment of preclinical AD cases in the *APOE* ε4^+^ SCD subgroup. Specifically, our hypothesis is that the rate of genuine AD cases contained in a study population might distort the correlation between Aβ-PET and plasma amyloid measurements.

We consider one of the main strengths of this study is that it includes a well-defined homogeneous population putatively positioned at a very early stage of the disease. We know that the main risk factors such as age and *APOE* do not follow the correlation expected in advanced stages of AD [64], and we have previously reported [64] that the *APOE* ε4 genotype had significant effects on the association with FBB-PET global SUVR in SCD subjects. Thus, AD does not behave linearly, and it could be that the TP42/40 ratio behaves independently from *APOE* when positioned to the left of the disease continuum. Our data show that refraining from strict inclusion criteria, such as *APOE* ε4 positivity, will be important to avoid detection bias.

An important limitation of this study is the fact that the FBB-PET cut-off value for positivity is arbitrary in SCD populations. The global SUVR > 1.45 cut-off value has been calculated for dementia patients but perhaps it should be adjusted for populations with different degrees of cognitive impairment or even on different segments of the AD continuum. Another limitation is the small sample size which warrants independent replication. Although Fandos et al. [49] reported similar results from the AIBL dataset in cognitively healthy and SCD individuals [65], it would be interesting to repeat the same analysis by Aβ cluster and replicate our findings in a larger population with a higher rate of amyloid PET-positive individuals to improve discrimination and accuracy of the plasma amyloid cut-off point.

Future research should address whether the association between brain and plasma Aβ levels in SCD participants is able to discriminate those older adults who will experience a fast cognitive decline from those who will remain stable over time.

## Conclusion

The present data show an inverse association between plasma Aβ42/40 ratios and brain fibrillary Aβ deposition in SCD participants. Including the TP42/40 plasma ratio could help generate a more cost-effective recruitment strategy for clinical trials independent of the *APOE* genotype (reflecting the real diversity of the *APOE* genotype in preclinical AD) and reducing the associated costs of preselecting subjects using expensive imaging techniques.

## Additional files

Additional file 1: **Table S1.** Demographics and clinical characteristics of subjects studied (FACEHBI [29]) for FBB-PET status being positive > 1.45. (DOCX 31 kb)

Additional file 2: Plasma amyloid beta levels measured with ABtest for the FACEHBI samples. (XLSX 28 kb)

Additional file 3: **Figure S1.** A) Distribution of FBB-PET and plasma ratios. B) Shapiro-Wilk test for FBB-PET and plasma ratios. C) Log distributions FBB-PET and plasma ratios. D) Shapiro-Wilk test for logarithmic FBB-PET and log-plasma ratios. A, B) Distributions and Shapiro-Wilk test for plasma ratios and FBB-PET to test normality. C, D) Distributions and Shapiro-Wilk test for transformed to logarithmic plasma ratios and FBB-PET to test normality. (PDF 299 kb)

Additional file 4: **Table S2.** Exploratory analysis. (DOCX 23 kb)

Additional file 5: **Table S3.** Regression analyses between Aβ plasma ratios and FBB-PET SUVR. (DOCX 18 kb)

Additional file 6: **Table S4.** ANOVAs comparing APOE ε4 carriers vs noncarriers. (DOCX 14 kb)

Additional file 7: **Figure S4.** *APOE* and plasma Aβ ratios. The effects of *APOE* genotype on plasma Aβ levels using ANOVA between *APO*E ε4 carriers and noncarriers in a boxplot representation with outlier analysis. (PDF 93 kb)

Additional file 8: **Figure S2.** Scatter plots for FBB-PET global SUVR and Aβ plasma ratios in SCD subjects. Correlations between plasma biomarkers and brain Aβ burden. Biomarkers values plotted against SUVR values from FBB-PET imaging: FP42/40 (A), BP42/40 (B), FP42/TP42 (C), and FP40/TP40 (D). (PDF 286 kb)

Additional file 9: **Figure S3.** Linear regression between FBB-PET and Aβ TP42/40 plasma ratio in *APOE* ε4 stratification SCD population. A) *APOE* ε4 carriers; B) *APOE* ε4 noncarriers. (PDF 115 kb)

Additional file 10: **Table S5.** Interaction between *APOE* and L_TP42/40. (DOCX 14 kb)

Additional file 11: **Figure S5.** Box plots for TP40 and TP42 by FBB-PET global SUVR status in SCD subjects. (JPEG 53 kb)

## Abbreviations

ABtest: Araclon Biotech test
AD: Alzheimer’s disease
ANOVA: Analysis of variance
*APOE*: Apolipoprotein E
*APP*: Amyloid precursor protein
AUROC: Area under the receiver operating characteristic curve
Aβ: Amyloid beta
BP: Bound to plasma components
CDR: Clinical Dementia Rating
CI: Confidence interval
CSF: Cerebrospinal fluid
ELISA: Enzyme-linked immunosorbent assay
FACEHBI: Fundació ACE Healthy Brain Initiative
FBB: Florbetaben
FLAIR: Fast fluid-attenuated inversion recovery
FOV: Field of view
FP: Free plasma
FSL: FMRIB Software Library
HADS: Hospital Anxiety and Depression Scale
LLQ: Lower limit of quantification
MCI: Mild cognitive impairment
MFE: Memory Failures in Everyday Life Questionnaire
MMSE: Mini-Mental State Examination
MPRAGE: Magnetization-prepared rapid gradient-echo
MRI: Magnetic resonance imaging
NBACE: Fundació ACE Neuropsychological Battery
NPV: Negative predictive value
OR: Odds ratio
PET: Positron emission tomography
PPV: Positive predictive value
SCD: Subjective cognitive decline
SUVR: Standardized uptake value ratio
TE: Echo time
TI: Inversion time
TP: Total plasma
TR: Repetition time
ULQ: Upper limit of quantification

## References

[1] Izco M, et al. Changes in the brain and plasma Abeta peptide levels with age and its relationship with cognitive impairment in the APPswe/PS1dE9 mouse model of Alzheimer’s disease. Neuroscience. 2014. 10.1016/j.neuroscience.2014.01.003. 10.1016/j.neuroscience.2014.01.003 24447596

[2] Ballard C (2011). Alzheimer’s disease. Lancet.

[3] Ruiz A, et al. Blood amyloid beta levels in healthy, mild cognitive impairment and Alzheimer’s disease individuals: replication of diastolic blood pressure correlations and analysis of critical covariates. PLoS One. 2013. 10.1371/journal.pone.0081334. 10.1371/journal.pone.0081334 PMC3842353 24312290

[4] Soininen HS, Scbeltens P (1998). Early diagnostic indices for the prevention of Alzheimer’s disease. Ann Med.

[5] Monge-Argilés, J. A. *et al.* [Biomarkers in the cerebrospinal fluid of patients with mild cognitive impairment: a meta-analysis of their predictive capacity for the diagnosis of Alzheimer’s disease]. Rev Neurol 2010;50:193–200. 20198590

[6] Toledo JB, Shaw LM, Trojanowski JQ (2013). Plasma amyloid beta measurements—a desired but elusive Alzheimer’s disease biomarker. Alzheimers Res Ther.

[7] Aizenstein HJ (2008). Frequent amyloid deposition without significant cognitive impairment among the elderly. Arch Neurol.

[8] Dickerson, B. C., Wolk, D. A. and Alzheimer’s Disease Neuroimaging Initiative. MRI cortical thickness biomarker predicts AD-like CSF and cognitive decline in normal adults. Neurology 78**,** 84–90 (2012). 10.1212/WNL.0b013e31823efc6c PMC3466670 22189451

[9] Davatzikos C, Bhatt P, Shaw LM, Batmanghelich KN, Trojanowski JQ (2011). Prediction of MCI to AD conversion, via MRI, CSF biomarkers, and pattern classification. Neurobiol Aging.

[10] Mehta PD (2000). Plasma and cerebrospinal fluid levels of amyloid beta proteins 1-40 and 1-42 in Alzheimer disease. Arch Neurol.

[11] Chouraki V (2015). Plasma amyloid-β and risk of Alzheimer’s disease in the Framingham Heart Study. Alzheimers Dement.

[12] van Oijen M, Hofman A, Soares HD, Koudstaal PJ, Breteler MM (2006). Plasma Aβ1–40 and Aβ1–42 and the risk of dementia: a prospective case-cohort study. Lancet Neurol.

[13] Graff-Radford NR (2007). Association of low plasma Aβ42/Aβ40 ratios with increased imminent risk for mild cognitive impairment and Alzheimer disease. Arch Neurol.

[14] Lambert J-C (2009). Association of plasma amyloid with risk of dementia: the prospective Three-City Study. Neurology.

[15] Nakamura A (2018). High performance plasma amyloid-β biomarkers for Alzheimer’s disease. Nature.

[16] Cantero JL, Iglesias JE, Van Leemput K, Atienza M (2016). Regional hippocampal atrophy and higher levels of plasma amyloid-beta are associated with subjective memory complaints in nondemented elderly subjects. J Gerontol Ser A Biol Sci Med Sci.

[17] Hansson O (2010). Evaluation of plasma Abeta(40) and Abeta(42) as predictors of conversion to Alzheimer’s disease in patients with mild cognitive impairment. Neurobiol Aging.

[18] Lopez OL (2008). Plasma amyloid levels and the risk of AD in normal subjects in the Cardiovascular Health Study. Neurology.

[19] Lövheim H, et al. Plasma concentrations of free amyloid β cannot predict the development of Alzheimer’s disease. Alzheimers Dement. 2017. 10.1016/j.jalz.2016.12.004. 10.1016/j.jalz.2016.08.016 27693182

[20] Fonseca JAS, et al. Factors that predict cognitive decline in patients with subjective cognitive impairment. Int Psychogeriatrics. 2015;27(10):1671–7. 10.1017/S1041610215000356 25812703

[21] Reisberg B, Shulman MB, Torossian C, Leng L, Zhu W (2010). Outcome over seven years of healthy adults with and without subjective cognitive impairment. Alzheimers Dement.

[22] Reid LM, MacLullich AMJ (2006). Subjective memory complaints and cognitive impairment in older people. Dement Geriatr Cogn Disord.

[23] Ogata S, Hayashi C, Sugiura K, Hayakawa K (2015). Association between subjective memory complaints and impaired higher-level functional capacity in people aged 60 years or older. Arch Gerontol Geriatr.

[24] Kryscio RJ (2014). Self-reported memory complaints: implications from a longitudinal cohort with autopsies. Neurology.

[25] Petersen RC (2001). Practice parameter: early detection of dementia: mild cognitive impairment (an evidence-based review). Report of the Quality Standards Subcommittee of the American Academy of Neurology. Neurology.

[26] Rabin LA (2015). Subjective cognitive decline in older adults: an overview of self-report measures used across 19 international research studies. J Alzheimers Dis.

[27] Abdelnour C (2017). Impact of recruitment methods in subjective cognitive decline. J Alzheimers Dis.

[28] Rodríguez-Gómez O, Abdelnour C, Jessen F, Valero S, Boada M. Influence of sampling and recruitment methods in studies of subjective cognitive decline. J Alzheimers Dis. 2015. 10.3233/JAD-150189. 10.3233/JAD-150189 26402087

[29] Rodriguez-Gomez O, et al. FACEHBI: a prospective study of risk factors, biomarkers and cognition in a cohort of individuals with subjective cognitive decline. Study rationale and research protocols. 2016. 10.14283/JPAD.2016.122. 10.14283/jpad.2016.122 29186280

[30] Lozoya-Delgado P, Ruiz-Sánchez de León JM, Pedrero-Pérez EJ (2012). Validation of a cognitive complaints questionnaire for young adults: the relation between subjective memory complaints, prefrontal symptoms and perceived stress. Rev Neurol.

[31] Alegret M (2013). Cut-off scores of a Brief Neuropsychological Battery (NBACE) for Spanish individual adults older than 44 years old. PLoS One.

[32] Zigmond AS, Snaith RP (1983). The hospital anxiety and depression scale. Acta Psychiatr Scand.

[33] Alegret M (2012). Normative data of a brief neuropsychological battery for Spanish individuals older than 49. J Clin Exp Neuropsychol.

[34] Fischl B (2004). Automatically parcellating the human cerebral cortex. Cereb Cortex.

[35] Fischl B (2002). Whole brain segmentation: automated labeling of neuroanatomical structures in the human brain. Neuron.

[36] Jenkinson M, Bannister P, Brady M, Smith S (2002). Improved optimization for the robust and accurate linear registration and motion correction of brain images. Neuroimage.

[37] Jenkinson M, Smith S (2001). A global optimisation method for robust affine registration of brain images. Med Image Anal.

[38] Bahar-Fuchs A (2013). Prediction of amyloid-β pathology in amnestic mild cognitive impairment with neuropsychological tests. J Alzheimers Dis.

[39] Pesini P (2012). Reliable measurements of the β-amyloid pool in blood could help in the early diagnosis of AD. Int J Alzheimers Dis.

[40] Ossenkoppele R, et al. Prevalence of amyloid PET positivity in dementia syndromes: a meta-analysis. JAMA. 2015. 10.1001/jama.2015.4669. 10.1001/jama.2015.4669 PMC4517678 25988463

[41] Jansen WJ (2015). Prevalence of cerebral amyloid pathology in persons without dementia. JAMA.

[42] Jack CR (2014). Age-specific population frequencies of cerebral β-amyloidosis and neurodegeneration among people with normal cognitive function aged 50–89 years: a cross-sectional study. Lancet Neurol.

[43] Alegret M, et al. Concordance between subjective and objective memory impairment in volunteer subjects. J Alzheimer’s Dis. 2015. 10.3233/JAD-150594. 10.3233/JAD-150594 26444795

[44] Lui JK (2010). Plasma amyloid-β as a biomarker in Alzheimer’s disease: the AIBL study of aging. J Alzheimers Dis.

[45] Devanand DP (2011). Plasma A and PET PiB binding are inversely related in mild cognitive impairment. Neurology.

[46] Rembach A (2014). Changes in plasma amyloid beta in a longitudinal study of aging and Alzheimer’s disease. Alzheimers Dement.

[47] Rembach A (2013). Plasma beta-amyloid levels are significantly associated with a transition toward Alzheimer’s disease as measured by cognitive decline and change in neocortical amyloid burden. Alzheimers Dement.

[48] Janelidze S, et al. Plasma β-amyloid in Alzheimer’s disease and vascular disease. Sci Rep. 2016. 10.1038/srep26801. 10.1038/srep26801 PMC4886210 27241045

[49] Fandos N (2017). Plasma amyloid β 42/40 ratios as biomarkers for amyloid β cerebral deposition in cognitively normal individuals. Alzheimers Dement (Amst).

[50] Toledo JB (2011). Factors affecting Aβ plasma levels and their utility as biomarkers in ADNI. Acta Neuropathol.

[51] Blennow K, Hampel H, Weiner M, Zetterberg H (2010). Cerebrospinal fluid and plasma biomarkers in Alzheimer disease. Nat Rev Neurol.

[52] Snyder HM (2014). Developing novel blood-based biomarkers for Alzheimer’s disease. Alzheimers Dement.

[53] Mattsson N (2015). Revolutionizing Alzheimer’s disease and clinical trials through biomarkers. Alzheimers Dement (Amst).

[54] Lewczuk P (2010). Amyloid β peptides in plasma in early diagnosis of Alzheimer’s disease: a multicenter study with multiplexing. Exp Neurol.

[55] Mayeux R (2003). Plasma A[beta]40 and A[beta]42 and Alzheimer’s disease: relation to age, mortality, and risk. Neurology.

[56] Fagan AM (2006). Inverse relation between in vivo amyloid imaging load and cerebrospinal fluid Aβ_42_ in humans. Ann Neurol.

[57] Giedraitis V (2007). The normal equilibrium between CSF and plasma amyloid beta levels is disrupted in Alzheimer’s disease. Neurosci Lett.

[58] Willemse E, et al. How to handle adsorption of cerebrospinal fluid amyloid-β (1–42) in laboratory practice? Identifying problematic handlings and resolving the issue by use of the Aβ42/Aβ40 ratio. Alzheimers Dement. 2017. 10.1016/j.jalz.2017.01.010. 10.1016/j.jalz.2017.01.010 28222302

[59] Insel PS (2016). Assessing risk for preclinical β-amyloid pathology with APOE, cognitive, and demographic information. Alzheimers Dement (Amst).

[60] Mielke MM (2012). Indicators of amyloid burden in a population-based study of cognitively normal elderly. Neurology.

[61] Rowe CC (2010). Amyloid imaging results from the Australian Imaging, Biomarkers and Lifestyle (AIBL) study of aging. Neurobiol Aging.

[62] Swaminathan S (2014). Association of plasma and cortical amyloid beta is modulated by APOE e4 status. Alzheimers Dement.

[63] Tateno, A., Sakayori, T. & Okubo, Y. The effect of apoe phenotype on the association of plasma beta-amyloid and cortical amyloid accumulation. AZ Kyoto 2017 at <http://www.adi2017.org/docs/default-source/default-document-library/adi_kyoto2017_englishabstractbook_online.pdf?sfvrsn=0>

[64] Moreno–Grau S, et al. Exploring APOE genotype effects on AD risk and β-amyloid burden in individuals with subjective cognitive decline: the FACEHBI study baseline results. Alzheimers Dement. 2017. 10.1016/j.jalz.2017.10.005. 10.1016/j.jalz.2017.10.005 29156223

[65] Ellis KA (2009). The Australian Imaging, Biomarkers and Lifestyle (AIBL) study of aging: methodology and baseline characteristics of 1112 individuals recruited for a longitudinal study of Alzheimer’s disease. Int. Psychogeriatrics.

