# Supplementary material for: Correlations between plasma and PET beta-amyloid levels in individuals with subjective cognitive decline: the Fundació ACE Healthy Brain Initiative (FACEHBI)
Source: Alzheimers Res Ther. 2018 Nov 29;10:119. doi: 10.1186/s13195-018-0444-1 (PMC6267075; doi:10.1186/s13195-018-0444-1)
Supplement: Supplementary file 1 — Table S1. Demographics and clinical characteristics of subjects studied (FACEHBI [29]) for FBB-PET status being positive > 1.45. (DOCX 31 kb) [file 13195_2018_444_MOESM1_ESM.docx]

**Table S1: *Demographics and clinical characteristics of subjects studied (FACEHBI***^29^***) for FBB-PET status* being positive >1.45 .**

| Variable | **+Aβ PET** | **-Aβ PET** |
| --- | --- | --- |
| Subjects, n | 18 | 182 |
| Age, years | 69.73 (4.92) | 65.41 (7.26) |
| Education. years | 13.33 (4.85) | 14.87 (4.71) |
| Gender (% males) | 55.6 | 35.7 |
| *APOE* (% e4 allele carriers) | 61.1 | 22.0 |
| Creatinine (mg/dl) | 0.96 (0.13) | 0.92 (0.15) |
| Body Mass Index (BMI, kg/m2) | 25.43 (2.79) | 26.76 (4.44) |
| Hematocrit (%) | 44.03 (3.69) | 43.06 (5.05) |
| FBB-PET SUVR | 1.62 (0.14) | 1.18 (0.15) |
| FP42/40 | 0.04(0.04) | 0.04 (0.03) |
| TP42/40 | 0.08 (0.09) | 0.09 (0.06) |
| FP40/TP40 | 0.44 (0.04) | 0.44 (0.06) |
| BP42/40 | 0.11 (0.12) | 0.13 (0.09) |
| FP42/TP42 | 0.23 (0.10) | 0.24 (0.21) |

Data are shown as mean (SD) unless otherwise specified. PET, positron electronic tomography; FBB, florbetaben(18F); SUVR, standardized uptake value ratios; FP, level of free Aβ peptide in plasma; TP, levels of total Aβ peptide in plasma; BP, levels of Aβ peptide bound to other plasma components.
