# Supplementary material for: Correlations between plasma and PET beta-amyloid levels in individuals with subjective cognitive decline: the Fundació ACE Healthy Brain Initiative (FACEHBI)
Source: Alzheimers Res Ther. 2018 Nov 29;10:119. doi: 10.1186/s13195-018-0444-1 (PMC6267075; doi:10.1186/s13195-018-0444-1)
Supplement: Supplementary file 3 — Figure S1. A) Distribution of FBB-PET and plasma ratios. B) Shapiro-Wilk test for FBB-PET and plasma ratios. C) Log distributions FBB-PET and plasma ratios. D) Shapiro-Wilk test for logarithmic FBB-PET and log-plasma ratios. A, B) Distributions and Shapiro-Wilk test for plasma ratios and FBB-PET to test normality. C, D) Distributions and Shapiro-Wilk test for transformed to logarithmic plasma ratios and FBB-PET to test normality. (PDF 299 kb) [file 13195_2018_444_MOESM3_ESM.pdf]

A) *Distribution FBB-PET and Plasma ratios*

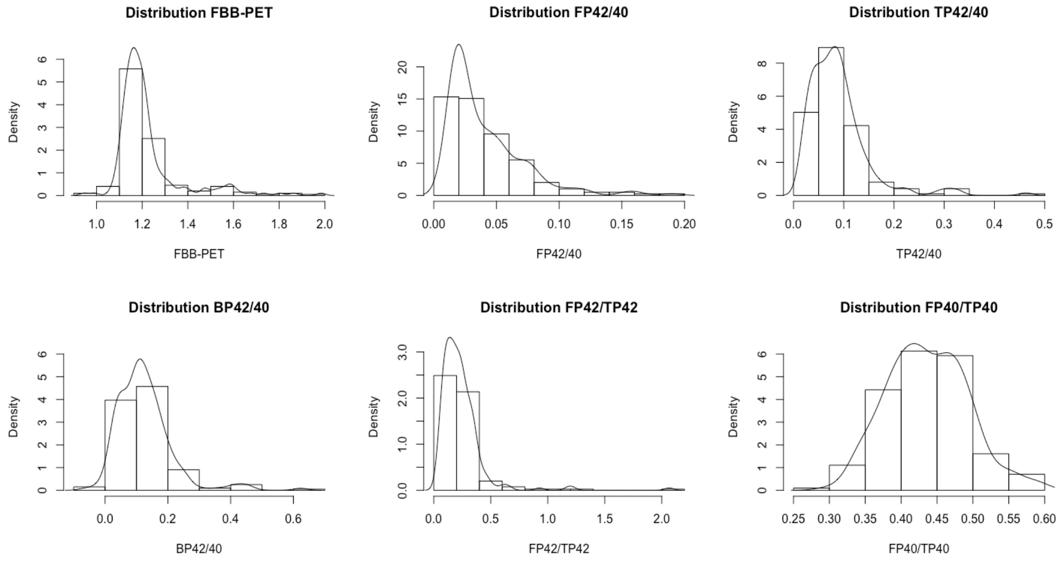

B) *Shapiro-Wilk test to FBB-PET and Plasma ratios*

Shapiro-Wilk	FBB_PET	FP42/40	TP42/40	BP42/40	FP42/TP42	FP40/TP40
W	0.717	0.799	0.794	0.861	0.605	0.991
p-value	<2.2E-16	2.76E-15	1.85E-15	1.59E-12	2.2E-16	0.243

C) Log *distributions FBB-PET and Plasma ratios*

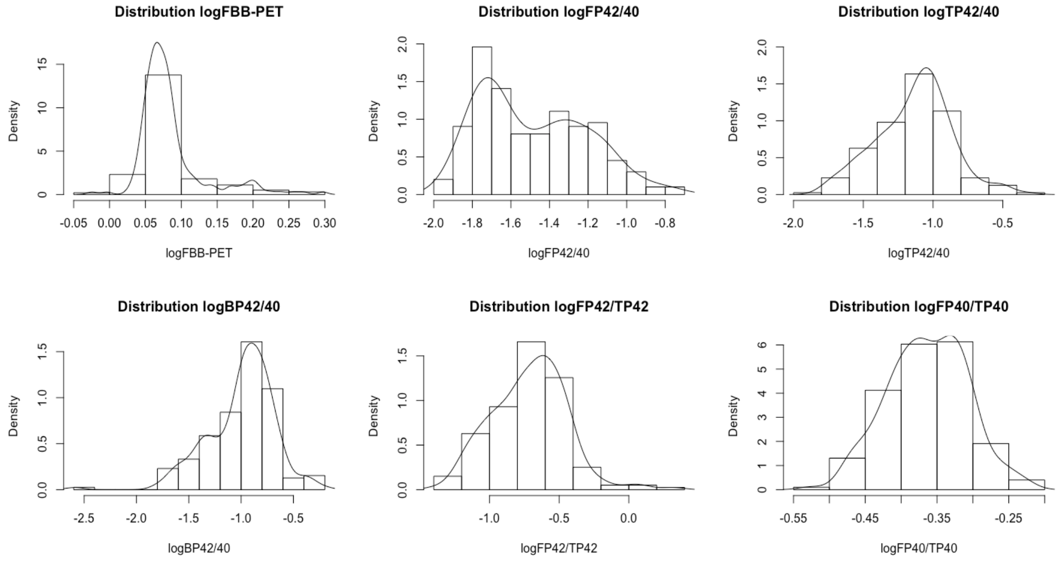

D) *Shapiro-Wilk test to Logarithmic FBB-PET and logPlasma ratios*

**Shapiro-Wilk**	**LogFBB_PET**	**LogFP42/40**	**LogTP42/40**	**LogBP42/40**	**LogFP42/TP42**	**LogFP40/TP40**
**W**	0.778	0.951	0.987	0.950	0.981	0.994
**p-value**	4.3E-16	2.22E-06	0.063	2.15E-06	0.008	0.666
