# Supplementary material for: Correlations between plasma and PET beta-amyloid levels in individuals with subjective cognitive decline: the Fundació ACE Healthy Brain Initiative (FACEHBI)
Source: Alzheimers Res Ther. 2018 Nov 29;10:119. doi: 10.1186/s13195-018-0444-1 (PMC6267075; doi:10.1186/s13195-018-0444-1)
Supplement: Supplementary file 4 — Table S2. Exploratory analysis. (DOCX 23 kb) [file 13195_2018_444_MOESM4_ESM.docx]

**Table S2: Exploratory analysis**

1. Pearson’s correlation between plasma ratios

|  | | **TP42/40** | **BP42/40** | **FP42/TP42** | **FP40/TP40** |
| --- | --- | --- | --- | --- | --- |
| **FP42/40** | Pearson’s r | 0.694  <2.2E-16  (0.614-0.760) | 0.533  5.4E-16  (0.425-0.626) | 0.338  1.1E-06  (0.209-0.456) | -0.082  0.248  (-0.219-0.057) |
|  | p-value |  |  |  |  |
|  | CI (95%) |  |  |  |  |
| **TP42/40** | Pearson’s r | - | 0.972  <2.2E-16  (0.963-0.979) | -0.271  1.06E-04  (-0.395- -0.137) | -0.159  8.77E-04  (-0.361- .0.098) |
|  | p-value |  |  |  |  |
|  | CI (95%) |  |  |  |  |
| **BP42/40** | Pearson’s r | - | - | -0.427  3.17E-10  (-0.534- -0.306) | -0.162  0.022  (-0.294- -0.023) |
|  | p-value |  |  |  |  |
|  | CI (95%) |  |  |  |  |
| **FP42/TP42** | Pearson’s r | - | - | - | 0.304  1.26E-05  (0.172-0.425) |
|  | p-value |  |  |  |  |
|  | CI (95%) |  |  |  |  |

1. Spearman correlation between plasma ratios

|  | | **TP42/40** | **BP42/40** | **FP42/TP42** | **FP40/TP40** |
| --- | --- | --- | --- | --- | --- |
| **FP42/40** | Spearman’s rho  p-value | 0.542  <2.2E-16 | 0.362  1.4E-07 | 0.447  3.59E-11 | -0.029  0.683 |
| **TP42/40** | Spearman’s rho  p-value | - | 0.964  <2.2E-16 | -0.412  1.53E-09 | -0.151  0.033 |
| **BP42/40** | Spearman’s rho  p-value | - | - | -0.546  <2.2E-16 | -0.07  0.360 |
| **FP42/TP42** | Spearman’s rho  p-value | - | - | - | 0.300  1.7E-05 |

1. Correlation between direct Aβ plasma levels, Aβ plasma ratio transformations and log transformed FBB-PET SUVR

| **Pearson’s correlation** | | | | | | | |
| --- | --- | --- | --- | --- | --- | --- | --- |
| **L_PET** | | | **FP42/40** | **TP42/40** | **BP42/40** | **FP42/TP42** | **FP40/TP40** |
| (n=199) | Pearson’s r | | -.109 | -.159^*^ | -.156^*^ | .055 | .084 |
|  | p-value (2-tailed) | | .126 | .025 | .027 | .437 | .238 |
|  | CI (95%) | | [-0.244 - 0.031] | [-0.291- -0.02] | [-0.289 - -0.017] | [-0.085 - 0.193] | [-0.056 - 0.22] |
| **Dichotomic** | | | | | | | |
| (n=199) | Pearson’s r | | -.152^*^ | -.264^**^ | -.243^**^ | .119 | .061 |
|  | p-value (2-tailed) | | .032 | 1.68E-04 | .001 | .095 | .391 |
|  | CI (95%) | | [-0.326- -0.058] | [-0.389- -0.129] | [-0.369- -0.107] | [-0.021 - 0.254] | [-0.079 - 0.198] |
| **Quartile** | | | | | | | |
| (n=199) | Pearson’s r | | -.196^**^ | -.252^**^ | -.230^**^ | .108 | .066 |
|  | p-value (2-tailed) | | .006 | 3.32E-04 | .001 | .128 | .355 |
|  | CI (95%) | | [-0.326- -0.058] | [-0.378- -0.117] | [-0.357- -0.094] | [-0.032 - 0.243] | [-0.074 - 0.203] |
| **Direct Aβ plasma** | | | | | | | |
| **(n=199) L_PET** | | FP40 | TP40 | FP42 | TP42 | BP40 | BP42 |
| Pearson’s r | | .098 | .048 | -.078 | -.117 | .007 | -.116 |
| p-value (2-tailed) | | .171 | .499 | .276 | .099 | .919 | .102 |
| CI (95%) | | [-0.042 - 0.234] | [-0.092 - 0.186] | [-0.215- 0.062] | [-0.252 - 0.023] | [-0.132 - 0.146] | [-0.251 -0.024] |

| **Spearman’s Rho** | | | | | | | |
| --- | --- | --- | --- | --- | --- | --- | --- |
| **L_PET** | | | **FP42/40** | **TP42/40** | **BP42/40** | **FP42/TP42** | **FP40/TP40** |
| (n=199) | Spearman’s rho | | -.165* | -.271** | -.264** | .113 | .136 |
|  | p-value | | .020 | 1.12E-04 | 1.7E-04 | .112 | .056 |
| **Dichotomic** | | | | | | | |
| (n=199) | Spearman’s rho | | -.144* | -.266** | -.272** | .132 | .106 |
|  | p-value | | .042 | 1.5E-04 | 1.03E-04 | .063 | .135 |
| **Quartile** | | | | | | | |
| (n=199) | Spearman’s rho | | -.174* | -.264** | -.254** | .107 | .102 |
|  | p-value | | .014 | 1.7E-04 | 3E-04 | .131 | .153 |
| **Logarithmic** | | | | | | | |
| (n=199) | Spearman’s rho | | -.165* | -.271** | -.262** | .113 | .134 |
|  | p-value | | .020 | 1.1E-04 | 2.2E-04 | .112 | .056 |
| **Direct Aβ plasma** | | | | | | | |
| **L_PET** | | **FP40** | **TP40** | **FP42** | **TP42** | **BP40** | **BP42** |
| Spearman’s rho | | .083 | .009 | -.110 | -.227** | -.065 | -.241** |
| p-value | | .243 | .899 | .121 | .001 | .364 | 5.9E-04 |

For the exploratory analysis, first we performed a Pearson and Spearman’s correlation between all plasma ratios to avoid collinearity (**A, B**). Next, we conducted a correlation between log transformed FBB-PET and plasma Aβ 42/40 ratios directly or transformed (dichotomic, with regard to the median of the population; quartile; and logarithmic). Bonferroni correction was used to adjust for multiple comparisons (< 1,92E-03). Abbreviations: FP, free plasma; TP, total plasma; BP, bound plasma; L_PET, logarithmic transformed positron electronic tomography score. (*p-value ≤ 0.05, **p-value ≤ 0.01).
