# Supplementary material for: Correlations between plasma and PET beta-amyloid levels in individuals with subjective cognitive decline: the Fundació ACE Healthy Brain Initiative (FACEHBI)
Source: Alzheimers Res Ther. 2018 Nov 29;10:119. doi: 10.1186/s13195-018-0444-1 (PMC6267075; doi:10.1186/s13195-018-0444-1)
Supplement: Supplementary file 5 — Table S3. Regression analyses between Aβ plasma ratios and FBB-PET SUVR. (DOCX 18 kb) [file 13195_2018_444_MOESM5_ESM.docx]

**Table S3. Regression analyses between Aβ plasma ratios and FBB-PET SUVR**

|  | **Unstardardized Coeficients** | | **Stardardized Coeficients** | | **CI 95.0%** | |
| --- | --- | --- | --- | --- | --- | --- |
|  | **B** | **Std. Error** | **Beta** | **Sig.** | **Lower bound** | **Upper bound** |
| **(Constant)** | -.059 | .036 |  | -1.655 | .100 | .011 |
| **Education** | -0.001 | 0.001 | -0.005 | 0.422 | -.002 | .001 |
| **Age** | 0.001 | 4.42E-04 | 0.216 | 0.001** | .001 | .002 |
| **Gender** | 0.009 | 0.006 | 0.092 | 0.167 | .022 | .049 |
| ***APOE*** | 0.035 | 0.007 | 0.330 | 6.95E-07** | -.004 | .021 |
| **L_TP42/40** | -0.041 | 0.011 | -0.239 | 2.59E-04** | -.063 | -.019 |
| R^2^$R^{2}$ **= 0.193, error =0.42** | | | | | | |
| **(Constant)** | -.043 | .038 |  | .258 | -.117 | .031 |
| **Education** | -.001 | .001 | -.077 | .274 | -.002 | .001 |
| **Age** | .001 | 4.42E-04 | .215 | .002* | .001 | .002 |
| **Gender** | .035 | .007 | .328 | 1.41E-06** | .021 | .049 |
| ***APOE*** | .007 | .007 | .071 | .297 | -.006 | .020 |
| **L_FP42/40** | -.023 | .011 | -.142 | .033 | -.045 | -.002 |
| R^2^ = **0.156, error =0.43** | | | | | | |
| **(Constant)** | -.044 | .035 |  | .212 | -.113 | .025 |
| **Education** | -.001 | .001 | -.057 | .418 | -.002 | .001 |
| **Age** | .001 | 4.42E-04 | .213 | .002* | .001 | .002 |
| **Gender** | .036 | .007 | .337 | 5.92E-07** | .022 | .050 |
| ***APOE*** | .009 | .006 | .097 | .148 | -.003 | .022 |
| **L_BP42/40** | -.032 | .010 | -.217 | .001** | -.051 | -.013 |
| R^2^ $R^{2}$**=**$R^{2}$ **0.183, error =0.42** | | | | | | |
| **(Constant)** | .005 | .034 |  | .875 | -.062 | .073 |
| **Education** | -.001 | .001 | -.074 | .296 | -.002 | .001 |
| **Age** | .001 | 4.44E-04 | .212 | .002* | 4.99E-04 | .002 |
| **Gender** | .036 | .007 | .335 | 9.11E-07** | .022 | .050 |
| ***APOE*** | .011 | .007 | .110 | .112 | -.002 | .024 |
| **L_FP42/TP42** | .020 | .012 | .113 | .094 | -.003 | .042 |
| R^2^ = **0.148, error =0.43** | | | | | | |
| **(Constant)** | .013 | .037 |  | .727 | -.060 | .085 |
| **Education** | -.001 | .001 | -.076 | .283 | -.002 | .001 |
| **Age** | .001 | 4.52E-04 | .223 | .002* | .001 | .002 |
| **Gender** | .034 | .007 | .324 | 2.58E-06** | .020 | .049 |
| ***APOE*** | .010 | .007 | .100 | .145 | -.003 | .023 |
| **L_FP40/TP40** | .070 | .057 | .083 | .224 | -.043 | .183 |
| R^2^$R^{2}$ **= 0.142, error =0.43** | | | | | | |

Linear regression analysis for all Aβ plasma ratios adjusting for age, gender, education and *APOE*; statistical significance was set to p<1.92E-03 to account for Bonferroni correction for multiple comparisons. (*p-value ≤ 0.01; **p-value ≤ 0.001).
