# Supplementary material for: Correlations between plasma and PET beta-amyloid levels in individuals with subjective cognitive decline: the Fundació ACE Healthy Brain Initiative (FACEHBI)
Source: Alzheimers Res Ther. 2018 Nov 29;10:119. doi: 10.1186/s13195-018-0444-1 (PMC6267075; doi:10.1186/s13195-018-0444-1)
Supplement: Supplementary file 6 — Table S4. ANOVAs comparing APOE ε4 carriers vs noncarriers. (DOCX 14 kb) [file 13195_2018_444_MOESM6_ESM.docx]

**Table S4. ANOVAs comparing APOE ε4 carriers vs non-carriers**

| **Aβ Plasma** | **P** | **F** | ${}^{\boldsymbol{2}}$ | **CI (95%)** | |
| --- | --- | --- | --- | --- | --- |
| TP42 | 0.680 | 0.170 | 0.001 | 0E-14 | 0.026 |
| TP40 | 0.628 | 0.236 | 0.001 | 0E-14 | 0.029 |
| TP42/40 | 0.768 | 0.087 | 4.41E-04 | 0E-14 | 0.019 |
| FP42/40 | 0.599 | 0.278 | 0.001 | 0E-14 | 0.030 |
| BP42/40 | 0.948 | 0.004 | 2.13E-05 | 0E-14 | 0.001 |
| FP42/TP42 | 0.386 | 0.754 | 0.004 | 0E-14 | 0.039 |
| FP40/TP40 | 0.041 | 4.249 | 0.021 | 0E-14 | 0.075 |

P for Bonferroni correction was 0.007.
