# Supplementary figures and images for: Correlations between plasma and PET beta-amyloid levels in individuals with subjective cognitive decline: the Fundació ACE Healthy Brain Initiative (FACEHBI)
Source: Alzheimers Res Ther. 2018 Nov 29;10:119. doi: 10.1186/s13195-018-0444-1 (PMC6267075; doi:10.1186/s13195-018-0444-1)

APOE and beta-amyloid plasma ratios

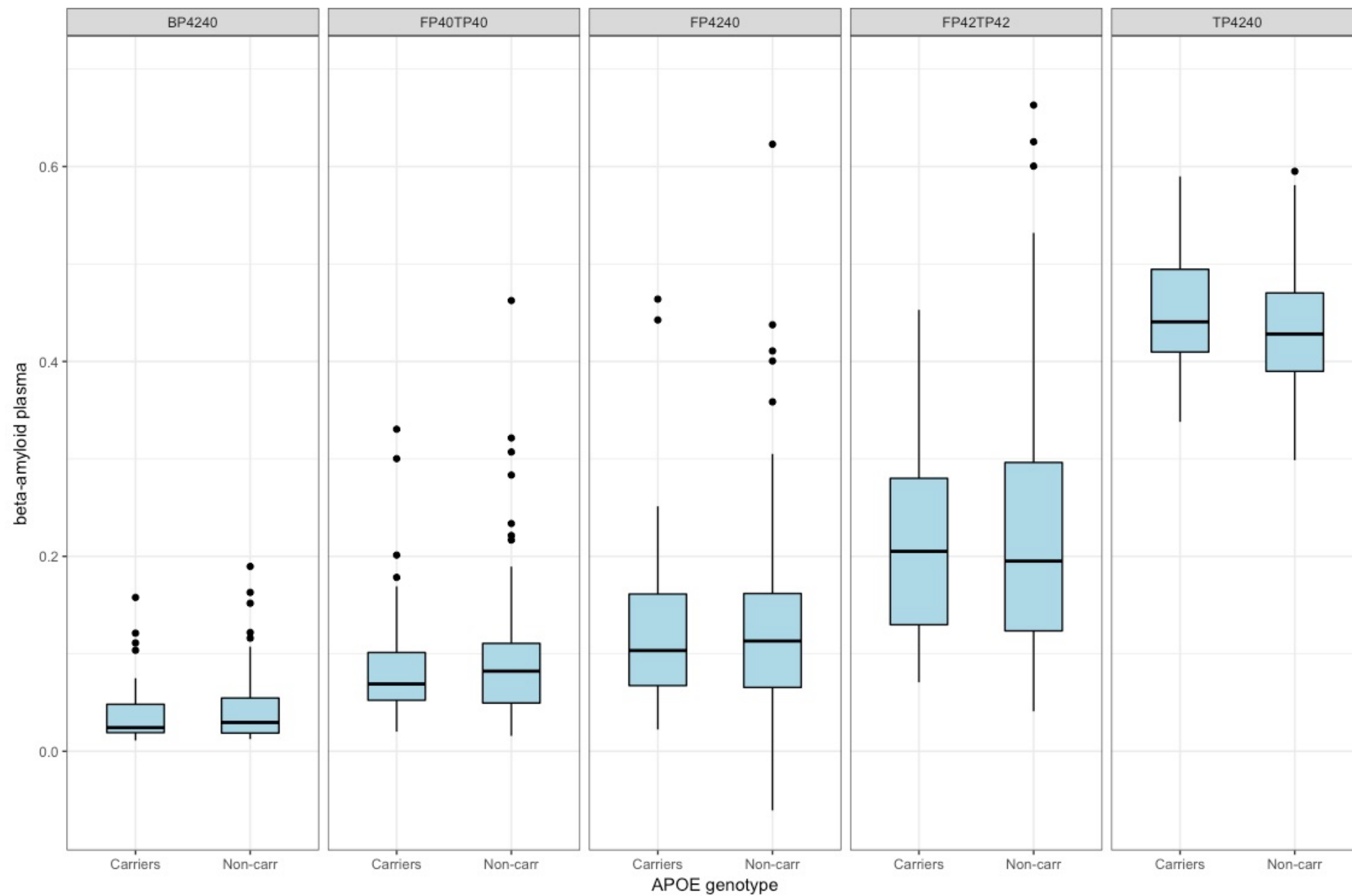

Supplement: Supplementary file 7 — Figure S4. APOE and plasma Aβ ratios. The effects of APOE genotype on plasma Aβ levels using ANOVA between APOE ε4 carriers and noncarriers in a boxplot representation with outlier analysis. (PDF 93 kb) [file 13195_2018_444_MOESM7_ESM.pdf]

A

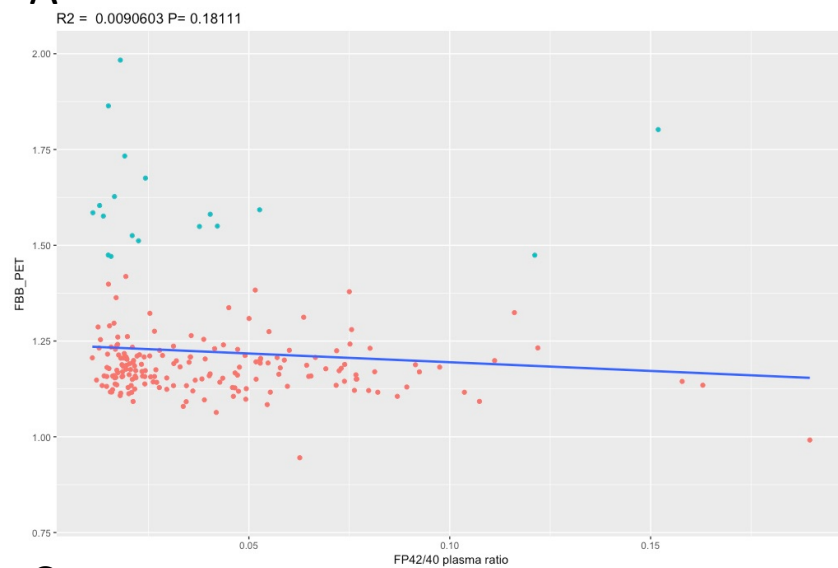

B

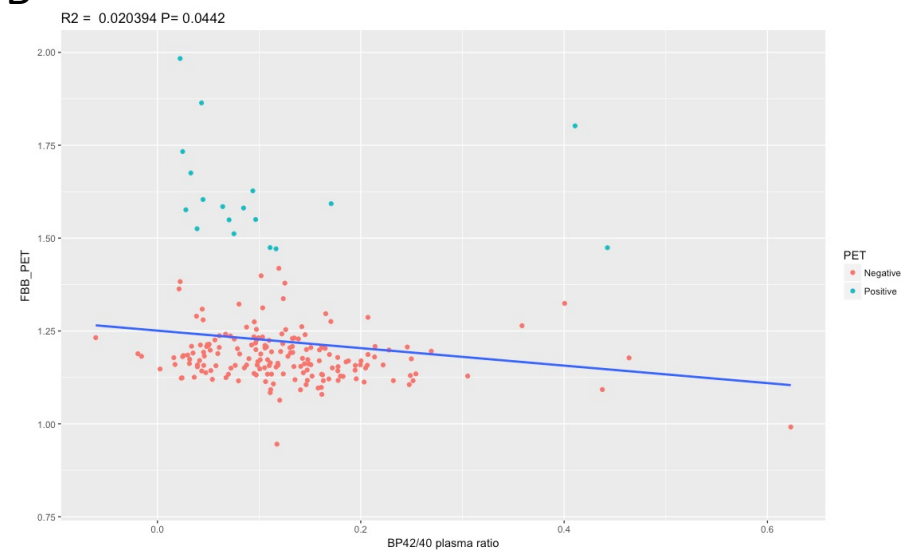

C

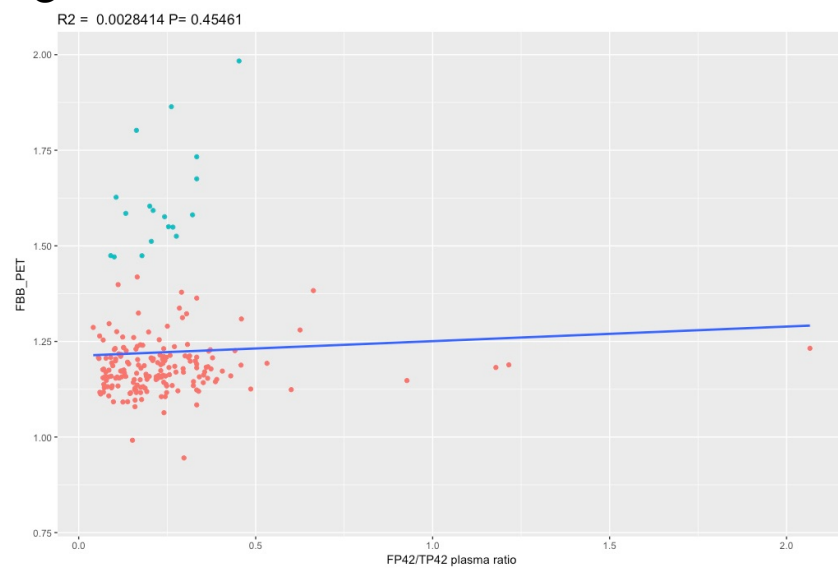

D

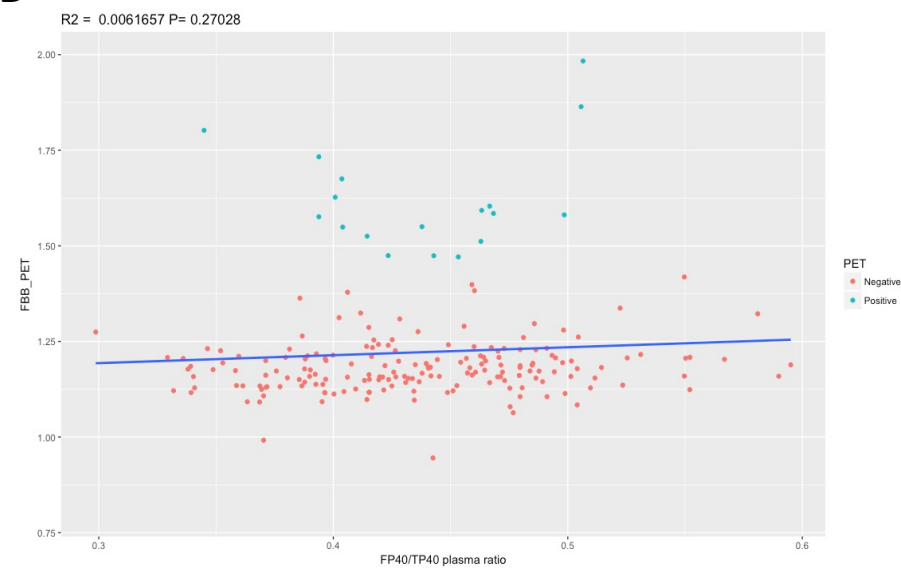

Supplement: Supplementary file 8 — Figure S2. Scatter plots for FBB-PET global SUVR and Aβ plasma ratios in SCD subjects. Correlations between plasma biomarkers and brain Aβ burden. Biomarkers values plotted against SUVR values from FBB-PET imaging: FP42/40 (A), BP42/40 (B), FP42/TP42 (C), and FP40/TP40 (D). (PDF 286 kb) [file 13195_2018_444_MOESM8_ESM.pdf]

**A** $R^2 = 0.099379$   $P = 0.024242$ 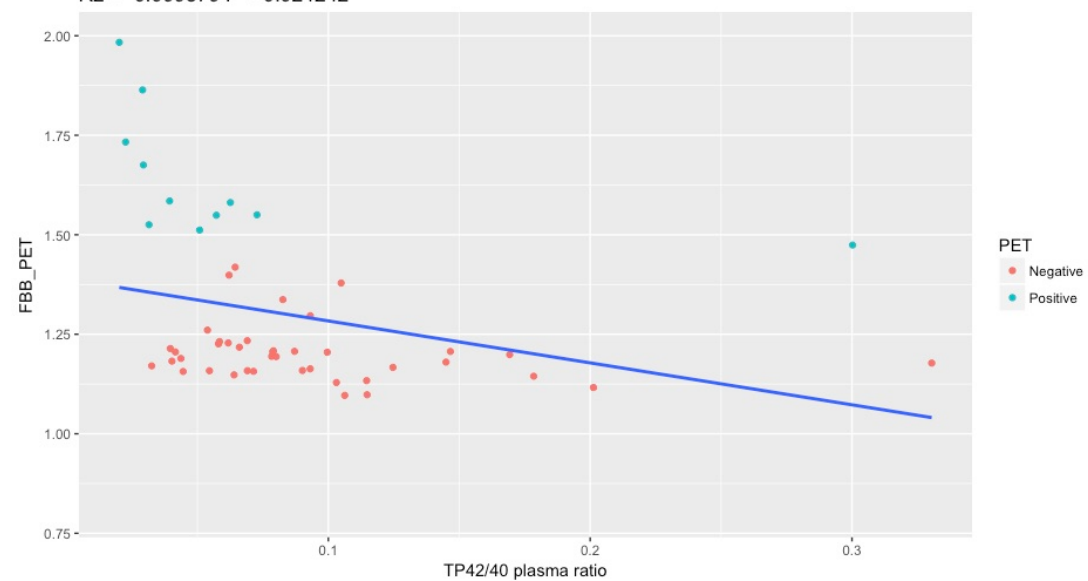**B** $R^2 = 0.0020218$   $P = 0.58737$ 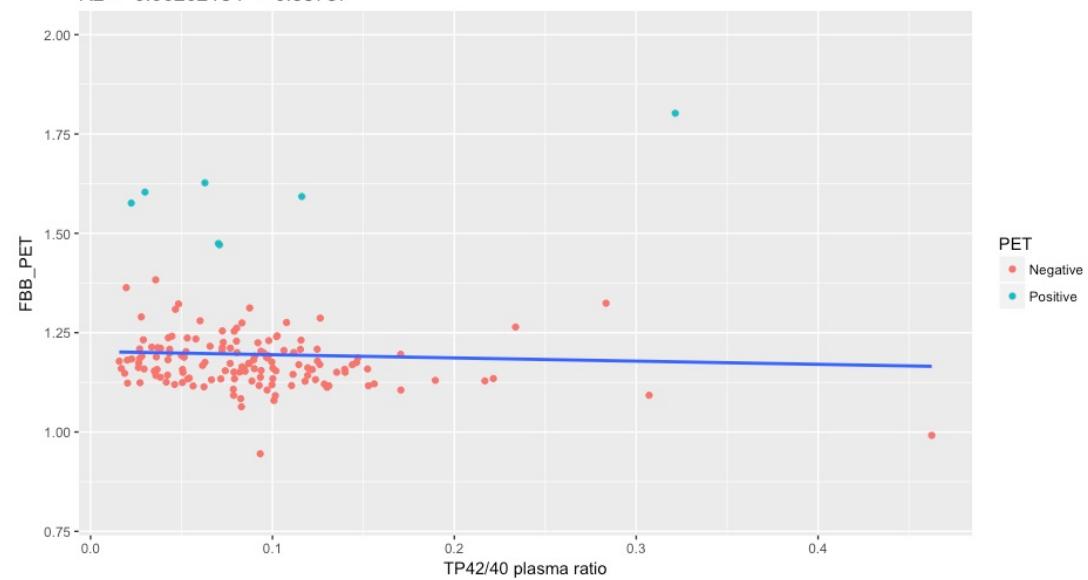

Supplement: Supplementary file 9 — Figure S3. Linear regression between FBB-PET and Aβ TP42/40 plasma ratio in APOE ε4 stratification SCD population. A) APOE ε4 carriers; B) APOE ε4 noncarriers. (PDF 115 kb) [file 13195_2018_444_MOESM9_ESM.pdf]

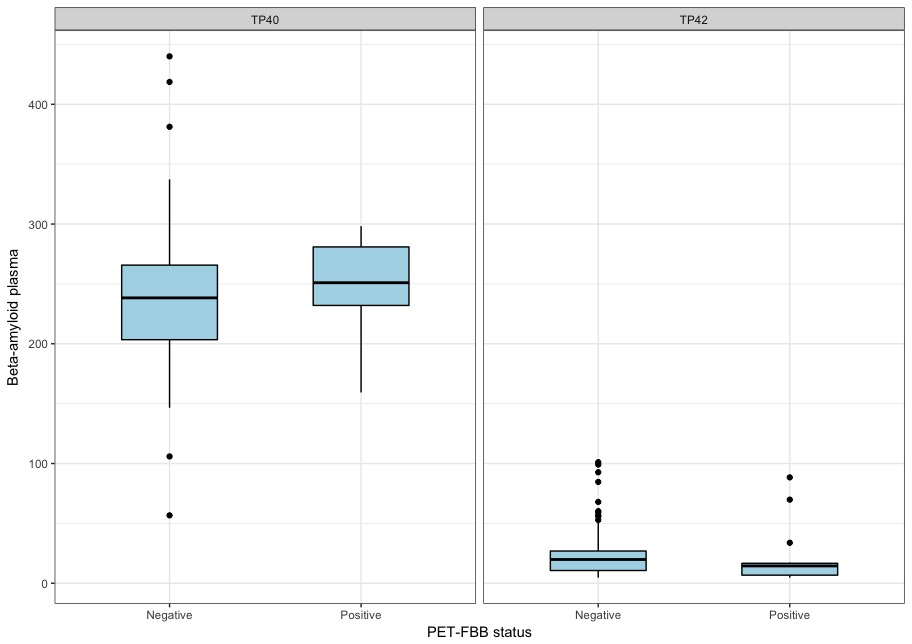

Supplement: Supplementary file 11 — Figure S5. Box plots for TP40 and TP42 by FBB-PET global SUVR status in SCD subjects. (JPEG 53 kb) [file 13195_2018_444_MOESM11_ESM.jpeg]
