# Supplementary material for: Correlations between plasma and PET beta-amyloid levels in individuals with subjective cognitive decline: the Fundació ACE Healthy Brain Initiative (FACEHBI)
Source: Alzheimers Res Ther. 2018 Nov 29;10:119. doi: 10.1186/s13195-018-0444-1 (PMC6267075; doi:10.1186/s13195-018-0444-1)
Supplement: Supplementary file 10 — Table S5. Interaction between APOE and L_TP42/40. (DOCX 14 kb) [file 13195_2018_444_MOESM10_ESM.docx]

**Table S5. Interaction between *APOE* and L_TP42/40**

|  | **P** | **OR** | **CI (95%) OR** | |
| --- | --- | --- | --- | --- |
|  |  |  | Lower bound | Upper bound |
| Age | 0.014 | 1.107 | 1.021 | 1.200 |
| *APOE* | 0.356 | 0.067 | 2.14E-04 | 20.961 |
| L_TP4240 | 0.685 | 0.577 | 0.041 | 8.210 |
| *APOE* by L_TP4240 | 0.105 | 0.022 | 2.30E-04 | 2.201 |
| Constant | 0.002 | 2.56E-05 | - | - |

Logistic regression model 3 with a study of interaction between *APOE* genotype and TP42/40 were used to assess predictors of FBB-PET SUVR positivity (cutoff >1.45). Abbreviations: CI = confidence interval; OR = odds ratio.
